# Hydrothermal green synthesis of MoS_2_ nanosheets for pollution abatement and antifungal applications†

**DOI:** 10.1039/d1ra03815j

**Published:** 2021-07-13

**Authors:** Mengistu Mulu, Dharmasoth RamaDevi, Neway Belachew, K. Basavaiah

**Affiliations:** Department of Inorganic and Analytical Chemistry, Andhra University Visakhapatnam-530003 India; A.U. College of Pharmaceutical Sciences, Andhra University Visakhapatnam-530003 India; Department of Chemistry, Debre Birhan University Debre Berhan Ethiopia neway.du@gmail.com neway@dbu.edu.et

## Abstract

In this study, we report a green synthesis of MoS_2_ nanosheets (NSs) using a facile hydrothermal technique in the presence of l-cysteine. l-Cysteine can serve as a greener source of sulfur as well as a capping agent to help the growth of MoS_2_ nanosheets. The prepared materials were characterized by X-ray powder diffraction (XRD), scanning electron microscopy (SEM) with energy dispersive spectroscopy (EDS), electron transmission microscopy (TEM), X-ray photoelectron microscopy (XPS), and Brunauer, Emmett, and Teller (BET) analysis. The results showed that MoS_2_ NSs are of high crystallinity with a lattice spacing of 0.61 nm. The optical bandgap of MoS_2_ NSs nanosheets prepared using l-cysteine as a source of sulfur was found to be 1.79 eV. The photocatalytic degradation of MoS_2_ NSs towards methylene orange (MO) and rhodamine blue (RB) dyes under sunlight was found to be promising for practical applications. The fast kinetics of degradation of MO and RhB was observed over a wide range of pH range. Moreover, MoS_2_ NSs showed excellent antifungal activities against *Trichophyton mentagrophytes* and *Penicillium chrysogenum* fungus.

## Abstract 2

In this study, we report a green synthesis of MoS_2_ nanosheets (NSs) using a facile hydrothermal technique in the presence of l-cysteine.

## Graphical abstract

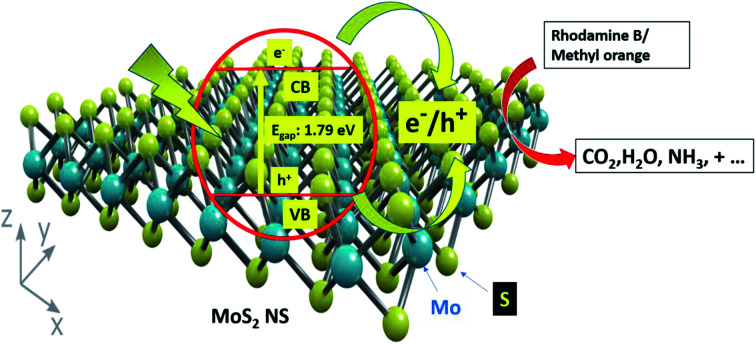

## 1. Introduction

During the last decades, the rapid increase in industrialization and human population has brought a multitude of contaminants to the aquatic environment. Synthetic organic dyes from dyeing industries make a substantial contribution.^1^ Due to this millions of people have been dying every year because of water pollution.^2^ Therefore, it is a prime concern to reduce or remove pollutants from their point source before releasing them into the environment. Adsorption, Fenton-oxidation, flocculation, and photocatalysis techniques have been widely used to abate toxic effluents.^3–5^ Photocatalytic degradation due to their efficiency can degrade pollutants without leaving unwanted residues.^6^ TiO_2_ and ZnO in this regard play a significant role in the harvesting of light for the photocatalytic degradation of organic compounds.^7^ However, inefficient utilization of light in the visible region and high electron–hole recombination rate prohibit its practical applications. Searching or modification of photocatalysts that working efficiently in the visible region is the prime concern in this topic.^8^ Composite with different sensitizers, doping of different elements and morphology optimization are common strategies for the modification of photocatalysts.^9^ More recently, the combination of photocatalyst with persistent luminescent materials as inner secondary light sources is introduced to enhance the photocatalytic efficiency of semiconductors.^10–12^

Two-dimensional (2D) functional nanostructured materials, such as graphene, hexagonal boron nitride (hBN), graphitic carbon nitride (g-C_3_N_4_) and metal dichalcogenides (MX_2_), owing to their inherent physicochemical properties, including high specific surface-to-volume ratio, anisotropy, chemical inertness, better charge carrier separation, the rich option of host–guest species, and excellent tribological efficiency, have gotten a lot of attention for photocatalytic applications.^13,14^ Moreover, they are widely used in a variety of fields, such as lubricants, energy storage, electrocatalysis, magnetoresistance, organic catalysis, sensing, and field transit.^14,15^

Recently, MX_2_-based transition metal chalcogenides (TMDs) where M (= Mo, W, *etc.*) is a transition metal of group VI and X (= S, Se, *etc.*) is a chalcogen that has been received a lot of attention owing to unique electrical, optical and mechanical properties. Among 2D nanomaterials, MoS_2_ due to intriguing physicochemical properties shows an application in antibacterial,^16,17^ biomedical,^18,19^ transistors,^20^ photodetectors,^21^ and, solar cells.^22^ Bulk MoS_2_ is an indirect bandgap semiconductor. However, as the number of layers is reduced to a few, bilayers, and even monolayers, the bandgap becomes direct.^23^ Such direct bandgap semiconductor materials are suitable for harvesting light. Hence, the synthesis of MoS_2_ with a few layered structures is a prime concern for nanosheets for photocatalytic applications such as dye degradation and solar energy conversion.^24,25^

MoS_2_ NSs with enhanced properties have been synthesised by various techniques, including chemical vapour deposition (CVD),^26^ thermal reductions,^27^ hydrothermal,^28^ laser ablation,^29^ Liquid Phase Exfoliation (LPE),^30,31^ and sol–gel methods.^32,33^ Despite the quality of MoS_2_ NSs synthesized by the aforementioned techniques, but they require highly sophisticated equipment and commonly used environmental malignant reagents. Most of the synthesis methods were used organic solvents, and surfactants during the synthesis of MoS_2_ NSs, which introduce toxicity to the environment and human health. Therefore, searching for kind reagents for the preparation of MoS_2_ NSs should require considerable attention. Biomolecule-assisted synthetic pathways have been a promising technique in recent years because they are greener and having suitable chemistry for the preparation of nanomaterials.

Green synthesised nanostructured materials play a significant role in practical applications, including medicinal and environmental. Biological ways of synthesizing nanoparticles using microorganisms, enzymes, fungi, and plants or plant extracts are eco-friendly green synthesis alternatives to chemical and physical methods.^18,19,34–36^ In this regard, amino acids due to interesting aqueous chemistry are widely applicable as a reducing agent, capping agent and a source of dopant atoms during the synthesis of nanomaterials.^37^ Among these, l-cysteine is a sulphur-containing derivative resulting from the oxidation of the side chains of cysteine amino acid thiol. It functions as an antioxidant, shielding tissues from radiation and toxins and thereby slowing the ageing process. l-Cysteine also reported as a sulphur source for the preparation of MoS_2_. For example, 1D CNTs–MoS_2_ hybrid materials synthesised by hydrothermal method using l-cysteine as sulphur soured for anodized materials in lithium-ion batteries.^38^ Similarly, Veeramalai *et al.*^39^ reported MoS_2_ layered structure by the reduction of MoO_3_ using l-cysteine as a sulphur source. The synthesised MoS_2_ showed high field performance applications. Despite the potential of l-cysteine as a source of sulphur and capping agent to protect an irreversible agglomeration, there is no comprehensive report on the facile hydrothermal synthesis and photocatalytic investigation towards dye degradation of MoS_2_ NSs.

Hence, we aimed to synthesis MoS_2_ NSs using a facile hydrothermal method. (NH_4_)_6_Mo_7_O_24_·4H_2_O salt and l-cysteine are the only precursors for the preparation of MoS_2_ NSs. The electronic, crystal phase formation, composition and surface morphology of the synthesised MoS_2_ nanosheet powder was investigated by UV-Vis, UV-DRS, XRD, FT-IR, XPS, TEM/SAED, and FE-SEM/EDX. The Brunauer–Emmett–Teller (BET) method used to determine the surface area analysis. The photocatalytic activities MoS_2_ NSs was investigated by the degradation of the two stable organic dyes, such as rhodamine B (RhB) and methylene orange. These two dyes commonly found in industrial wastewater and are extremely toxic to human health due to their solubility, carcinogenicity, and teratogenicity. Moreover, the antifungal activity of MoS_2_ was studied against *Penicillium chrysogenum* and *Trichophyton mentagrophytes* fungus.

## 2. Experimental

### 2.1. Materials

All of the chemicals used in this analysis were analytical grade levels from Sigma-Aldrich, and Himedia. All chemicals were directly used without further purification. Specifically, ammonium molybdate tetrahydrate [(NH_4_)_6_Mo_7_O_24_·4H_2_O], and l-cysteine (C_3_H_7_NO_2_S) were obtained from Himedia, India. Methylene orange (MO), and rhodamine blue (RhB) were supplied from Sigma-Aldrich, India. The ultrapure water (Milli-Q) was used as a solvent throughout the whole experiment.

### 2.2. Nanosheet MoS_2_ synthesis

**Fig. 1:**
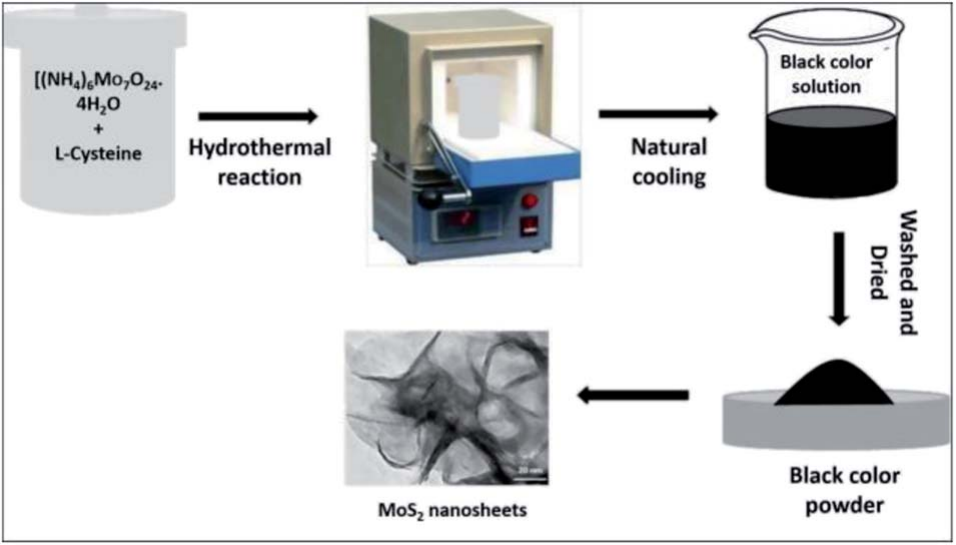
The schematic illustration for the hydrothermal synthesis of MoS_2_ NSs.

MoS_2_ NSs were synthesized by a facile and green hydrothermal synthesis approach using l-cysteine as a sulphur source. Particularly, 2.0 g (NH_4_)_6_Mo_7_O_24_·4H_2_O and 4.0 g l-cysteine were mixed in 50 mL deionized water. The solution was then sonicated for 30 minutes to create a clear solution. In a muffle furnace, the solution was poured into a 100 mL stainless steel autoclave and heated at 200 °C for 12 hours (Fig. 1). The autoclave was cooled to room temperature after the reaction had completed. Next, the black precipitate was centrifuged and thoroughly washed with water to remove unreacted residue and dried in the oven at 70 °C for 18 hours. The black powder, the as-synthesised MoS_2_ nanosheet, was kept for further use (ESI, Fig. 1†).

### 2.3. Characterizations

PANalytical Xpert Pro diffractometer with, Cu K_1_ = 0.154056 nm as a radiation source was used to record the X-ray diffraction (XRD) of MoS_2_ NSs. Transmission electron microscopy (TEM) images were recorded from FEI TECNAI G2 S-Twin, equipped with a 200 kV field emission gun (FEG). The field emission scanning electron microscopy (FE-SEM) images were obtained from JEOL, JSM-7600F SEM at an accelerated voltage of 0.1 to 30 kV equipped with X-ray dispersive spectroscopy (EDX). The chemical composition of the MoS_2_ NSs was detected by X-ray photoelectron spectroscopy (XPS, ESCALAB 250Xi). The Brunauer–Emmett–Teller (BET) (BELSORB-28SA/18SA/18PLUS) analyser studied the surface area and pore size from the N_2_ adsorption data.^40,41^

### 2.4. RhB and MO photocatalytic degradation

The photocatalytic activity of the MoS_2_ NSs towards the degradation of MO and RhB was investigated under natural sunlight irradiation. The adsorption–desorption equilibrium of dye was carried in the dark for 30 min before illumination. Specifically, 30 mg of MoS_2_ NSs was taken for 50 mL of 20 ppm dye solution. The sample was in dark to attain the adsorption–desorption and illuminate at midday (11 am to 3 pm) sunlight. At different time intervals, 3 mL of the solution took from the reaction vessel and centrifuged at 5000 rpm for 5 min to separate the catalyst. The residual concentration of MO and RhB in the solution was determined using a UV-visible spectrophotometer at *λ*_max_ = 500 nm and 553 nm, respectively. The quantitative explanation of adsorption in the dark and degradation under sunlight was calculated by eqn (1) and (2) respectively.

1 *q* = (*C*_0_ − *C*_t_) × *V*/*m*

2 Degradation (%) = (*C*_0_ − *C*_t_) × 100/*C*_0_ =

where *q* (mg g^−1^) the amount of MO adsorbed on the surface of MoS_2_ nanosheet; *V* (L) is the volume of the aqueous solution, *m* (g) is the mass of MoS_2_ nanosheet, *C*_0_ (mg L^−1^), the initial concentration of MO/RhB dye; *C*_t_ (mg L^−1^) the concentration of MO/RhB dye after adsorption/degradation. Furthermore, the antifungal activities of MoS_2_ NSs using Agar-Well diffusion method was further investigated against the *T. mentagrophytes* and *P. chrysogenum* fungus. The detailed protocol presents in ESI 1.†

## 3. Results and discussion

### 3.1. Characterizations of MoS_2_ nanosheets

**Fig. 2:**
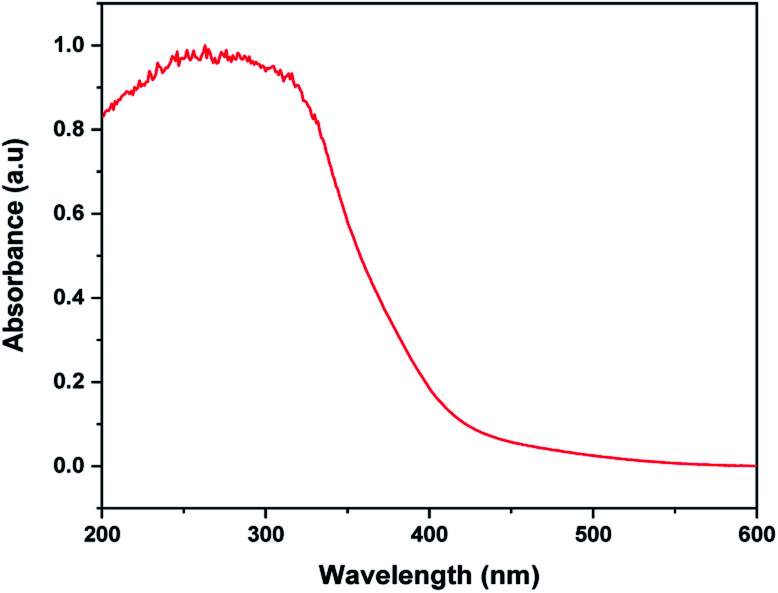
The UV-Vis absorption spectrum of MoS_2_ NSs.

**Fig. 3:**
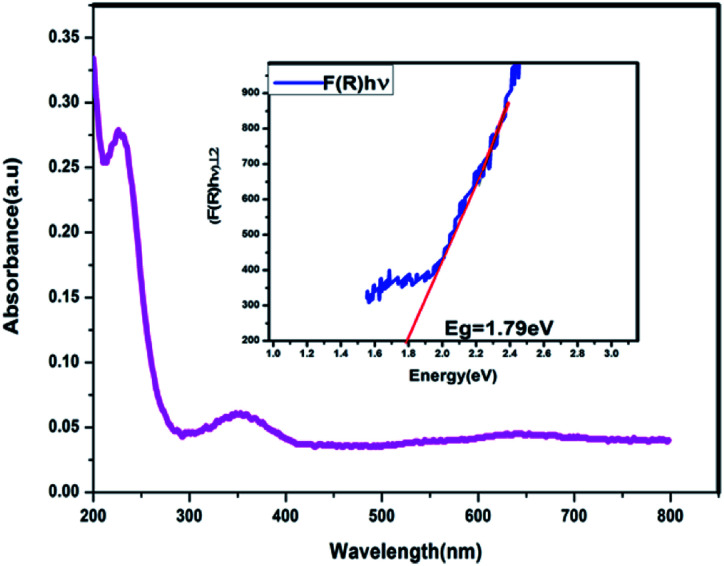
The UV-DRS absorption spectrum of MoS_2_ NSs.

The UV-Vis absorption spectrum of MoS_2_ NS was recorded to investigate the optical property. As shown in Fig. 2, the absorption edge of MoS_2_ NS extends to the visible region. Moreover, the optical bandgap energy of MoS_2_ NS was calculated from the UV-DRS spectrum (Fig. 3). The intrinsic direct bandgap of MoS_2_ NSs was found to 1.79 eV. This implies that the activation [formation of electron (e^−^) and holes (h^+^)] of MoS_2_ NSs is possible under the irradiation of the visible light region. Similar bandgap energy reported elsewhere.^34^

**Fig. 4:**
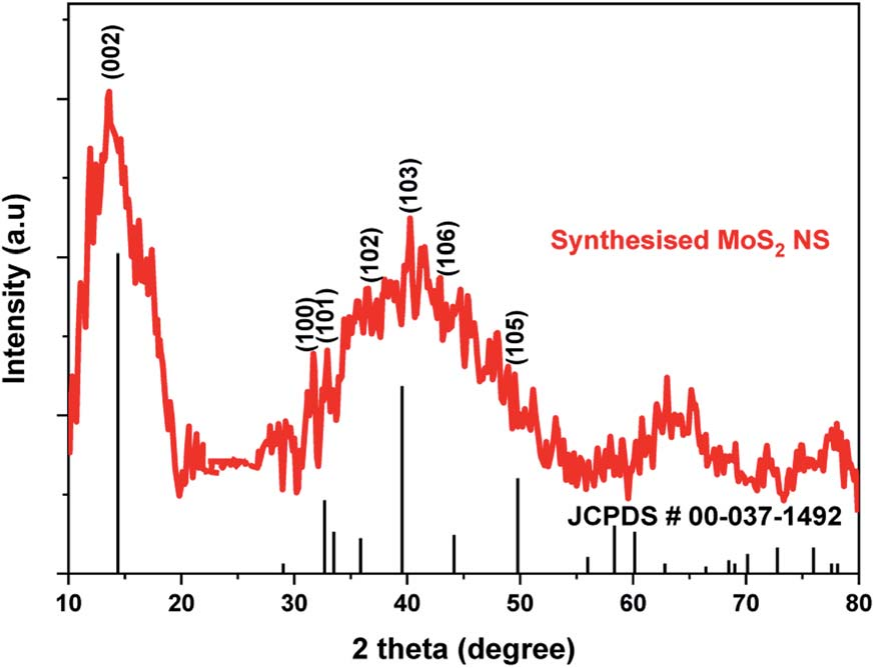
The XRD graph of MoS_2_ NSs.

Fig. 4 shows the XRD pattern for MoS_2_ NSs. The diffractogram confirms the formation of hexagonal MoS_2_ NSs from Mo^2+^ salt and l-cysteine as a sulfur source. The characteristic diffraction peak observed at 13.6°, 31.97°, 32.39°, 36.53°, 40.26°, 42.39°, and 48.94° which are indexed to (002), (100), (101), (102), (103), (106), and (105) planes of MoS_2_, respectively (JCPDS#: 00-037-1492).^42^ The broadened of the (002) peak and diffraction at a low angle (2*θ*: 13.6°) than bulk MoS_2_ (14.37°) shows the formation of few or single layers of MoS_2_ like graphene.^43^

**Fig. 5:**
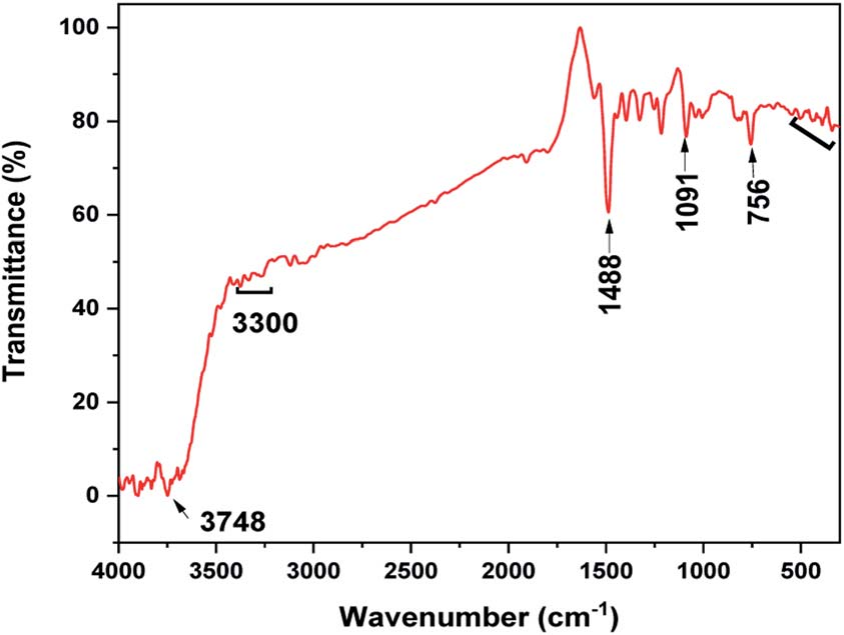
The FT-IR spectrum of MoS_2_ NSs.

The FTIR spectrum of the MoS_2_ NSs was recorded to investigate the composition of samples based on the vibrational properties (Fig. 5). The spectrum of MoS_2_ NSs shows the broad bands between 3748 and 3400 cm^−1^ attribute to the characteristics of O–H stretching of the intermolecular and intramolecular hydrogen bonds.^44,45^ The characteristic vibrational bands at 3300–2800 cm^−1^ and 1488 cm^−1^ can be assigned to the stretching of the C–H alkyl stretching band in l-cysteine.^46^ Thus, the FTIR results further affirmed the presence of residual amino acid on the surface of MoS_2_. The medium absorption bands observed at 756 cm^−1^, 1091 cm^−1^, and 1400 cm^−1^ are due to MoS_2_.^47,48^ Moreover, the band between 540 cm^−1^ and 400 cm^−1^ credit to the S–S bond vibrations.^47^

**Fig. 6:**
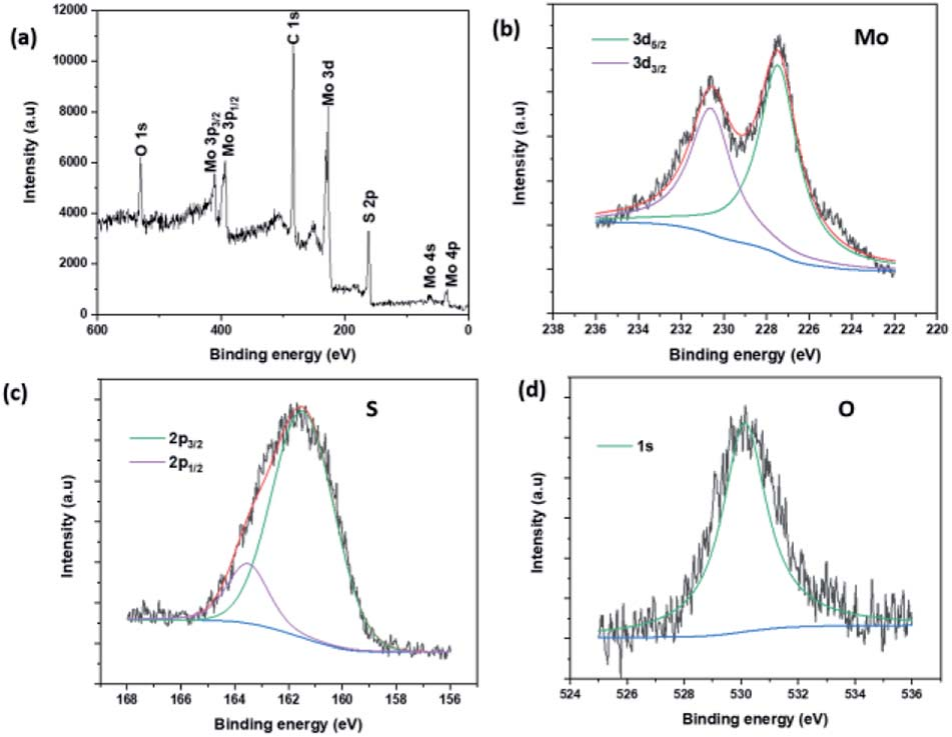
(a) The survey XPS spectrum of MoS_2_ NSs, (b–d) the high-resolution XPS spectra of Mo, S and O, respectively.

The composition and chemical state of MoS_2_ NSs was studied by X-ray photoelectron spectroscopy (XPS). Fig. 6a presents the survey scan XPS spectrum of MoS_2_ NS. The spectrum depicts the presence of peaks due to C_1s_ and O_1s_ at 285.49 eV and 533.07 eV respectively. These are due to the residual l-cysteine capped onto the MoS_2_ surface. The high-resolution peaks of Mo, S, and O are shown in Fig. 6b–d. The peak binding energies of Mo and S are agreed well with the theoretical binding energies of the corresponding orbital electrons of Mo and S elements. Further, both the Mo 3d_5/2_ (227.48 eV) and the Mo 3d_3/2_ (230.63 eV) features presented in Fig. 6b are deconvoluted with only one function, indicating the presence of only one molybdenum chemical species at the surface.^49,50^ Moreover, the binding energy shows a shift as compared to the elemental Mo peaks affirms the formation of Mo^4+^ chemical state.^39,51^ Likewise, a similar analysis was done on sulfur, obtained characteristic peaks at the binding energy of 161.58 eV and 162.78 eV due to the S 2p_3/2_, and assigned to S 2p_1/2_, respectively.^52,53^

**Fig. 7:**
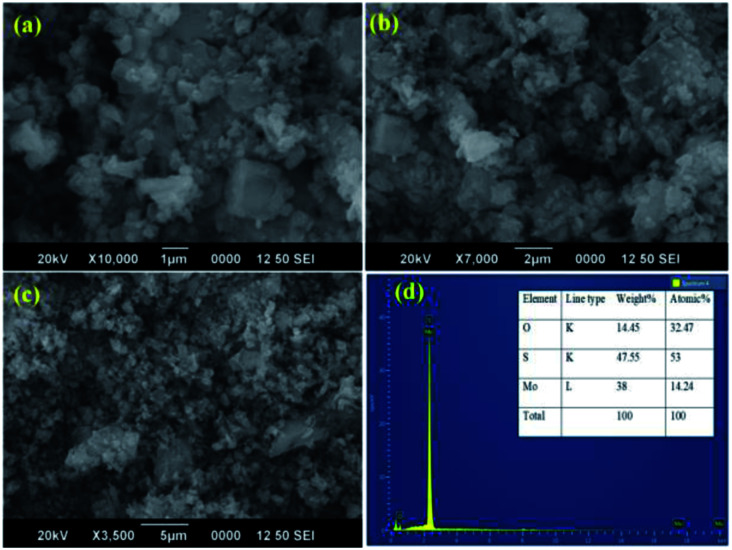
(a–c) The representative SEM images NSs at different magnifications, (d) EDS spectrum of MoS_2_.

**Fig. 8:**
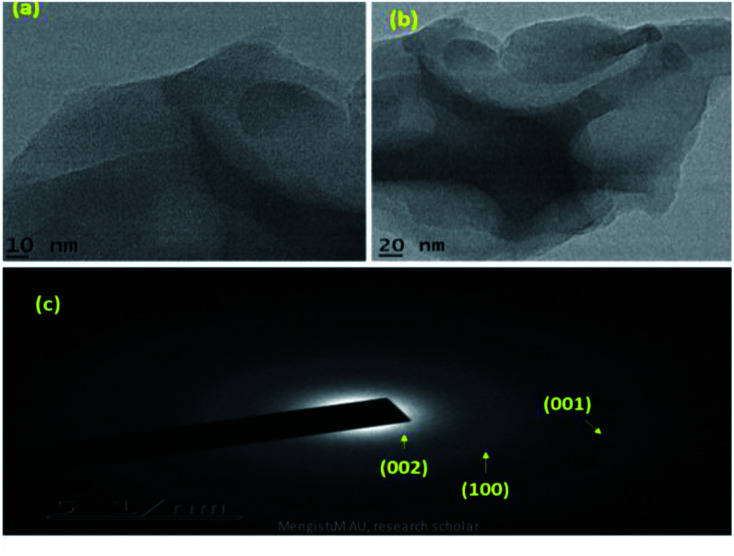
(a and b) The representative TEM images NSs at different magnifications, (c) SAED pattern of MoS_2_.

SEM and TEM microscopic techniques were used to investigate the surface morphology of MoS_2_ NSs. Fig. 7a–c shows the SEM image of the synthesized MoS_2_ NSs. The image shows an aggregated and rough surface morphology of MoS_2_ NSs. Interesting sheet-like morphology observed under TEM analysis (Fig. 8). As it is shown in Fig. 8a and b, a few layers and aggregated MoS_2_ were synthesized *via* a facile hydrothermal method. The selected area electron diffraction (SAED) pattern (Fig. 8c) of the MoS_2_ sheet shows the smoothness of the concentric circle, which indicates the poor crystallinity of the sheet. Moreover, the (002), (100), and (001) planes keep stable, indicating the good stability of MoS_2_ along this plane.

### 3.2. RhB and MO dye degradation *via* photocatalysis

**Fig. 9:**
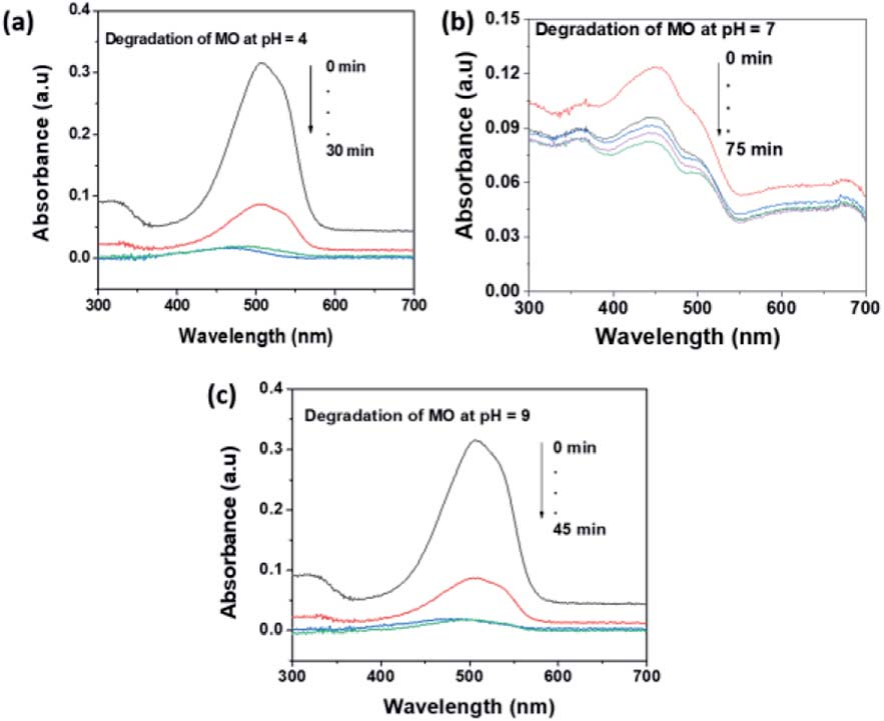
The effect of pH of solution for the degradation MO at (a) pH = 4, (b) pH = 7 and (c) pH = 9.

The pH of the solution an important factor that affects the surface charge of the photocatalyst and the charge of the organic dye. As a result, the electrostatic interaction between the dye molecule and catalyst surface is highly affected by the pH of the solution. Methyl orange (MO) and rhodamine B (RhB) dyes were selected as representative anionic and cationic dyes respectively, for the photocatalytic investigation of MoS_2_ NSs. The acidic (pH = 4), neutral (pH = 7) and basic (pH = 9) medium were optimized to study the pH effect. Fig. 9 shows the effect of pH on the photocatalytic degradation of MO. As can be seen in Fig. 9, MoS_2_ NSs (30 mg) show a strong potential to degrade MO (20 mg L^−1^) at a given pH of the solution. Still, degradation of MO shows fast kinetics and almost complete degradations at pH = 4 due to the protonated surface at low pH enhance the electrostatic interaction with the anionic dye. Alkaline pH (pH = 9) also suitable to get a complete degradation of MO slightly at a longer time (Fig. 9c) than acidic. The increase in hydroxyl ions concentration in the solution facilitated the formation of further hydroxyl radical; this could be plausible for an enhanced reaction rate of degradation under alkaline conditions. The neutral pH (pH = 7) shows a relatively poor rate of degradation and efficiency (72%) (Fig. 9b). Hence, the acid or alkaline medium is suggested to get a better photocatalytic degradation efficiency of MO.

**Fig. 10:**
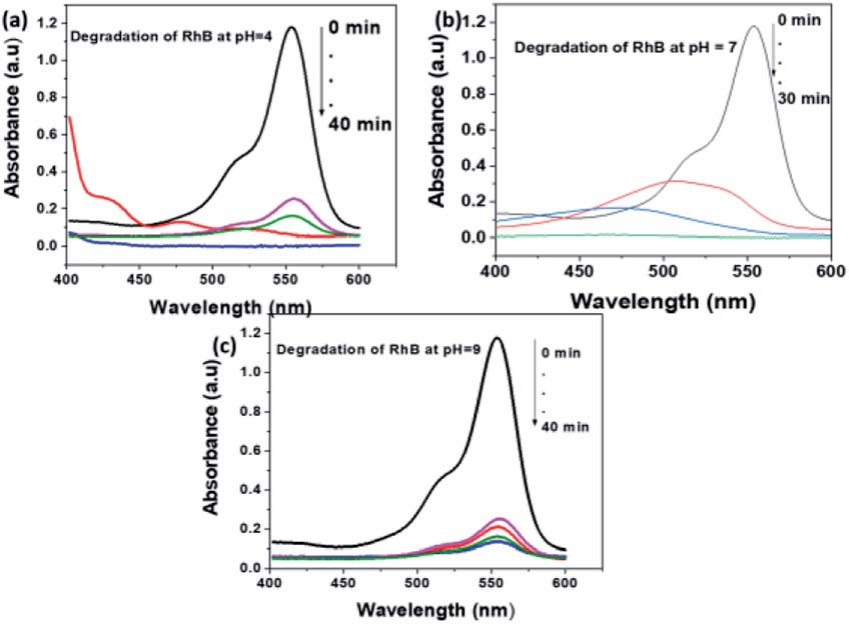
The effect of pH of solution for the degradation RhB at (a) pH = 4, (b) pH = 7 and (c) pH = 9.

A similar investigation on the degradation of RhB performed at various pH of the solution. The potential of MoS_2_ NSs also observes for the degradation of a stable RhB dye. As shown in Fig. 10, complete degradation of 50 mL RhB (20 mg L^−1^) using 80 mg MoS_2_ NSs at acid and neutral solutions. Similar justification on the effect of pH for the degradation of RhB is applied to as MO. RhB was successfully removed with a high rate of degradation was observed at neutral pH (Fig. 10b). Concomitantly, the absorption maxima of RhB at *λ*_max_ = 553 nm showed a shift to a lower wavelength region during the degradation process, which attributes to the formation of intermediate products in the meanwhile.

**Fig. 11:**
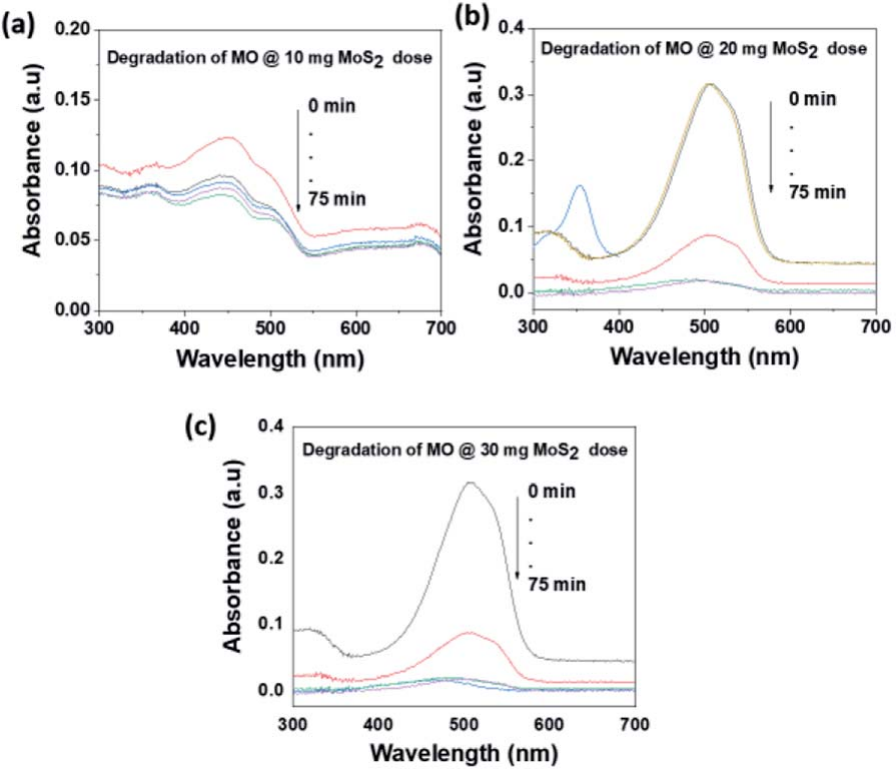
The effect of MoS_2_ NSs dose for the degradation MO.

**Fig. 12:**
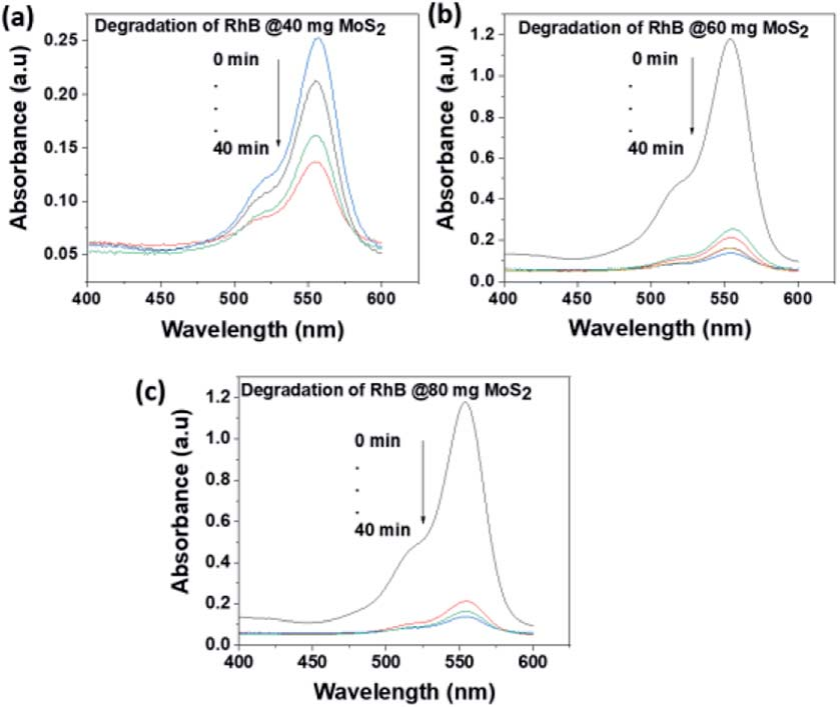
The effect of MoS_2_ NSs dose for the degradation RhB.

The photocatalytic activity of MoS_2_ NSs was further investigated at various doses of the catalyst. At a fixed amount of dye concentration (50 mL, 20 mg L^−1^), at neutral pH and room temperature. Fig. 11 shows the effect catalyst dose for the removal of MO at the various dose of MoS_2_ (10 mg, 20 mg, and 30 mg). Results showed that fast catalytic activity of MoS_2_ NSs observed as a dosage increases from 20 mg to 30 mg. However, yet there is no significant difference between 20 mg and 30 mg dosages, and therefore 20 mg of could be considered as optimum for 50 mL MO (20 mg L^−1^). The same analogy was observed for RhB degradation. As shown in Fig. 12, the degradation of RhB (50 mL, 20 mg L^−1^) was investigated at various doses of MoS_2_ NSs (40 mg, 60 mg, and 80 mg). The degradation efficiency increases with an increasing dose of MoS_2_ NSs. The maximum value was obtained at 80 mg MoS_2_ NSs dose, considered as optimum.

### 3.3. Photocatalytic mechanism

**Fig. 13:**
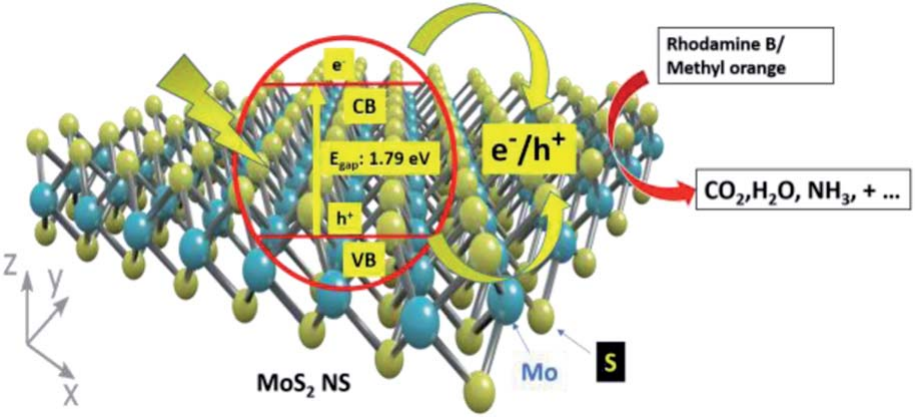
The plausible degradation mechanism of organic dye.

Fig. 13 shows the photocatalytic degradation mechanism of organic dye using catalysts. Firstly, the dye adsorbed on the surface of the MoS_2_ NSs in the dark because of the interaction between the shade and MoS_2_ NSs. Owing to the low bandgap of the MoS_2_ nanosheets, electrons and holes are generated. Electrons in the conduction band react with the available oxygen to form superoxide radicals (O_2_˙). Similarly, holes in the valence band react with water to form hydroxyl (OH˙) radicals. The formation of the OH˙ and O_2_˙ radicals has appeared. As a result, the generated OH radicals degrade the adsorbed dyes efficiently at room temperature by simple magnetic stirring. The production of radicals results in the mineralization of dyes into CO_2_, H_2_O, and other gaseous products.

### 3.4. Anti-fungal activity of MoS_2_ nanosheets

**Fig. 14:**
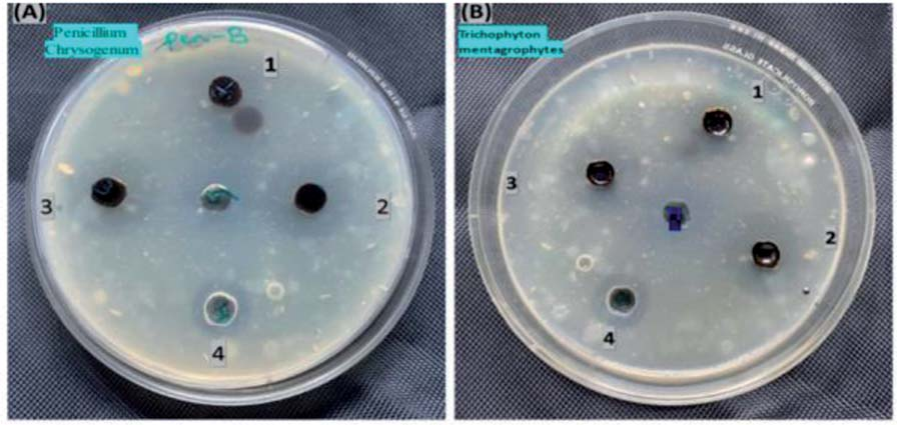
The antifungal activities of synthesized MoS_2_ nanosheet against (A) *Penicillium chrysogenum* and (B) *Trichophyton mentagrophytes*.

The antimicrobial activity of MoS_2_ NSs against standard strains of *Trichophyton mentagrophytes* and *Penicillium chrysogenum* presented in Fig. 14. The higher inhibition zone is observed in *Penicillium chrysogenum* with higher concentrations of the synthesized material, *i.e.*, 5 μg mL^−1^ of MoS_2_ nanosheet solutions. Mainly due to the increase in concentrations of MoS_2_ nanosheets, the diameter of the inhibition zone increased, *i.e.* 10 mm at 1 μg mL^−1^, 14 mm at 3 μg mL^−1^ and 16 mm at 5 μg mL^−1^. At higher concentrations, the diameter of the inhibition zone was more than the same concentrations of the standard drug inhibition (fluconazole (5 μg mL^−1^)). This result shows that a newly prepared nanosheet shows promising and requires further study for antifungal-related applications.

## 4. Conclusions

In conclusion, MoS_2_ NSs are synthesised successfully *via* a simple and green hydrothermal method using ammonium molybdenum hydrate and l-cysteine. In this process, to help the growth of MoS_2_ NSs, l-cysteine can be used as a sulfur source and a capping agent. The synthesized MoS_2_ NSS exhibited excellent photocatalytic degradation efficiency towards MO and RhB dyes under sunlight irradiation. It was achieved >99% degradation efficiency relatively fast rate reaction. The narrow bandgap (1.79 eV), and large surface area (6.46 m^2^ g^−1^) of the MoS_2_ NSs are responsible for the degradation of the dyes under sunlight. MoS_2_ NSs shows the toxic effect against fungi growth.

## Conflicts of interest

There are no conflicts to declare.

## Supplementary Material

RA-011-D1RA03815J-s001
