# Supplementary material for: Hydrothermal green synthesis of MoS2 nanosheets for pollution abatement and antifungal applications
Source: RSC Adv. 2021 Jul 13;11(40):24536–42. doi: 10.1039/d1ra03815j (PMC9036892; doi:10.1039/d1ra03815j)
Supplement: RA-011-D1RA03815J-s001 [file RA-011-D1RA03815J-s001.pdf]

Electronic Supplementary Material (ESI) for RSC Advances.
This journal is © The Royal Society of Chemistry 2021

Hydrothermal Green Synthesis of MoS_2 Nanosheets for Pollution Abatement and Antifungal Applications

Mengistu Mulu,[a] Dharmasoth RamaDevi,[b] Neway Belachew,[c,*] K. Basavaiah[a,*]

[a]Department of Inorganic and Analytical Chemistry, Andhra University, Visakhapatnam-530003, India.

[b]A.U. College of Pharmaceutical Sciences, Andhra University, Visakhapatnam-530003, India.

[c]Department of Chemistry, Debre Birhan University, Debre Berhan, Ethiopia.email: neway.du@gmail.com/neway@dbu.edu.et

SI. 1. Antifungal activities of MOS_2 nanoscheet.

The antifungal activity was carried out by employing 24 h cultures with given compounds by using the Agar-Well diffusion method. The medium (Potato Dextrose Agar) was sterilized by autoclaving at 120 °C (15 psi) for about 30min. About 20 ml of the medium was seeded with the respective strains of Fungi and transferred aseptically into each sterilized petri dish. The plates were left at room temperature for solidification. Each plate, a single well of 6 mm diameter was made using a sterile borer. The test compounds were freshly reconstituted with suitable solvents (distilled H_2O) and tested at various concentrations (1, 2 and 3). The samples and the control (distilled H_2O) along with standard (Fluconazole) were placed in 6 mm diameter well. Petri plates were incubated at 28 ± 2°C for about 24 hrs. Standard with a concentration of 5 µg/ml was used as a positive control.

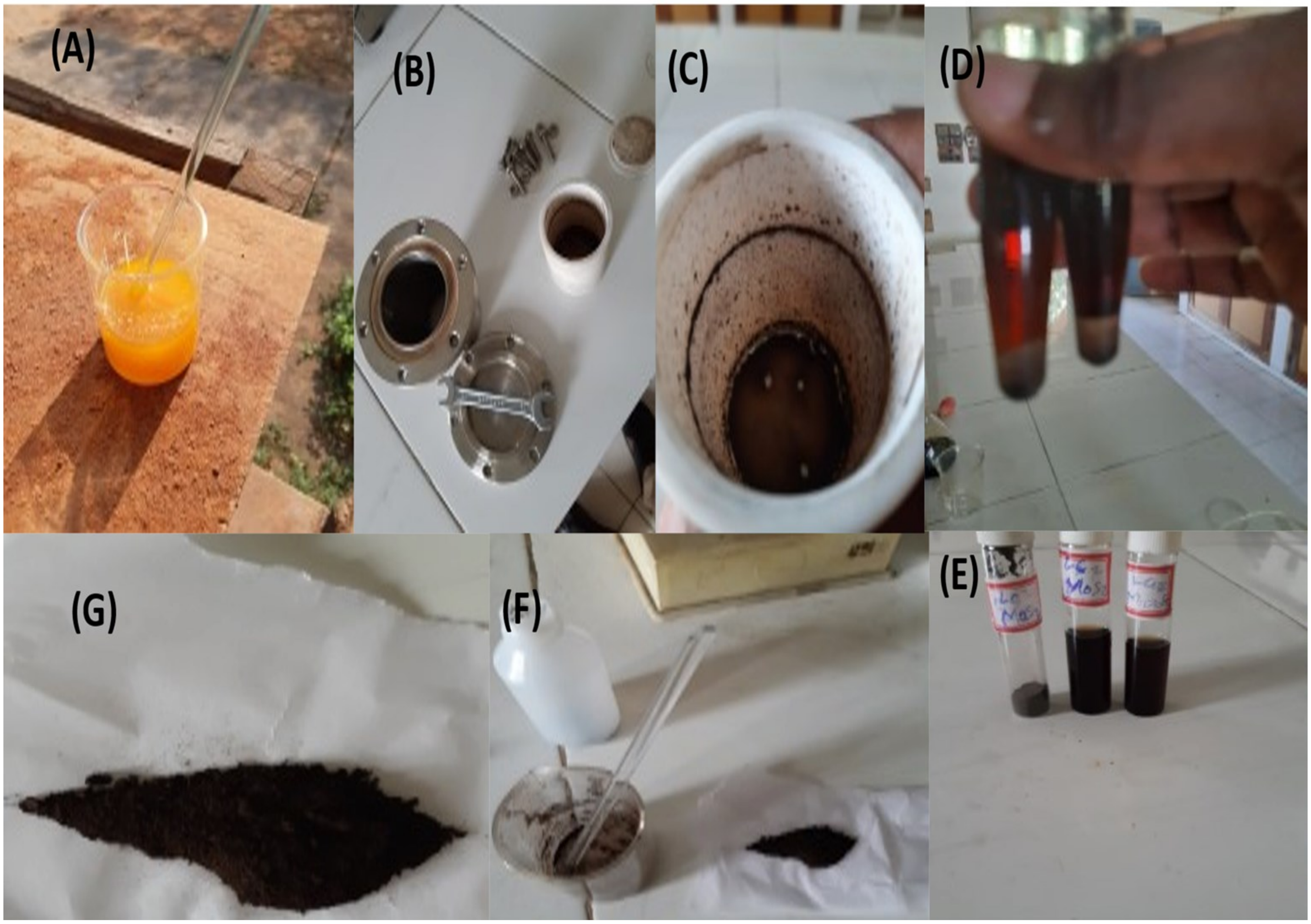

SI. Figure 1   Synthesis of MoS_2 nanosheets

SI.2. Surface area and pore size analysis

The surface area and pore size analysis of MoS_2 NSs were determined from the N_2 adsorption dat. The surface area of MoS_2 NSs was recorded using BET method (SI. Figure 2). The specific surface area of found to be was found to be 7.69 (m^2g^{-1}). Similarly, the mean pore diameter determined using BHJ plot (SI. Figure 3) was 1.2 nm. This moderate surface area and pore size of the photocatalyst enhance the adsorption of dye, which in turn facilitate the photocatalysis turn over.

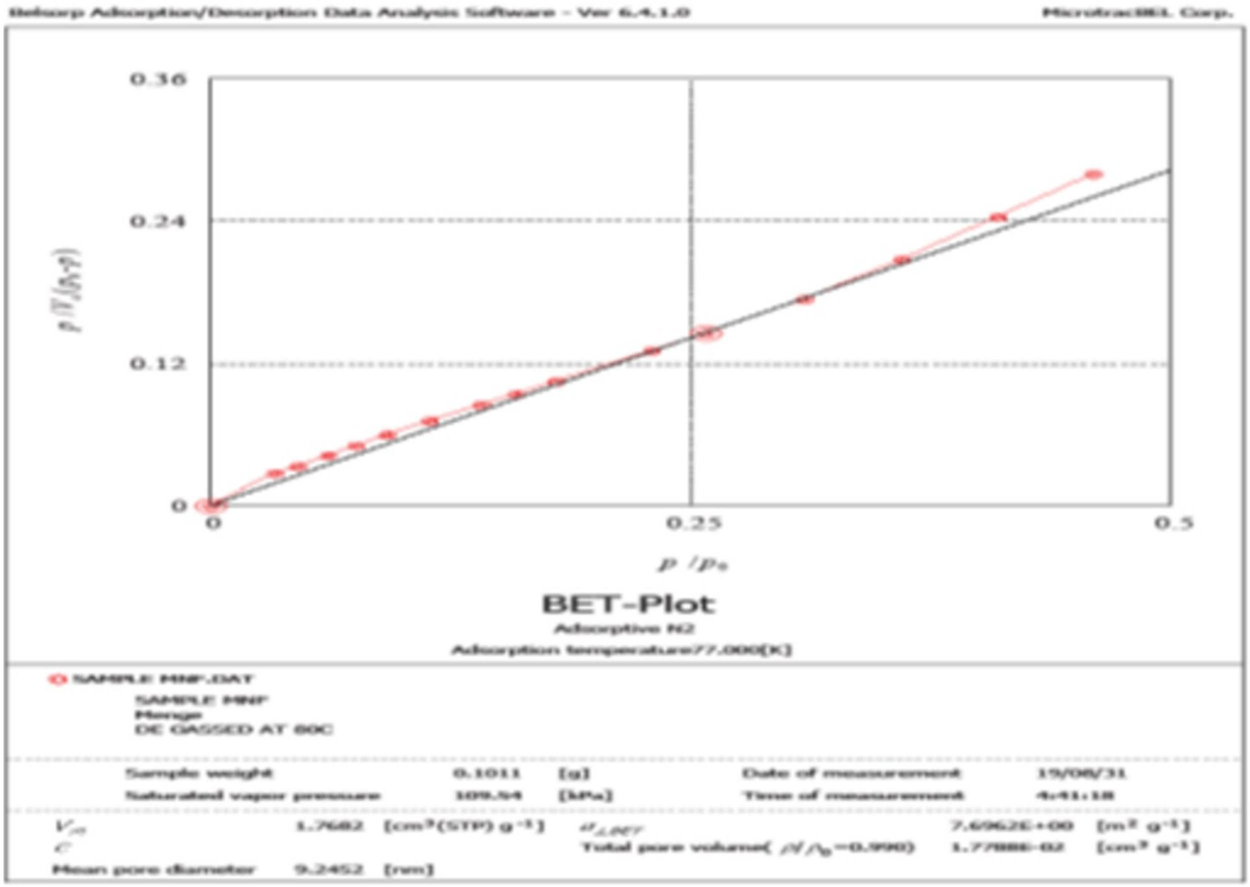

SI Figure 2 : The Brunauer-Emmett-Teller (BET) plot of MoS_2 nanosheet

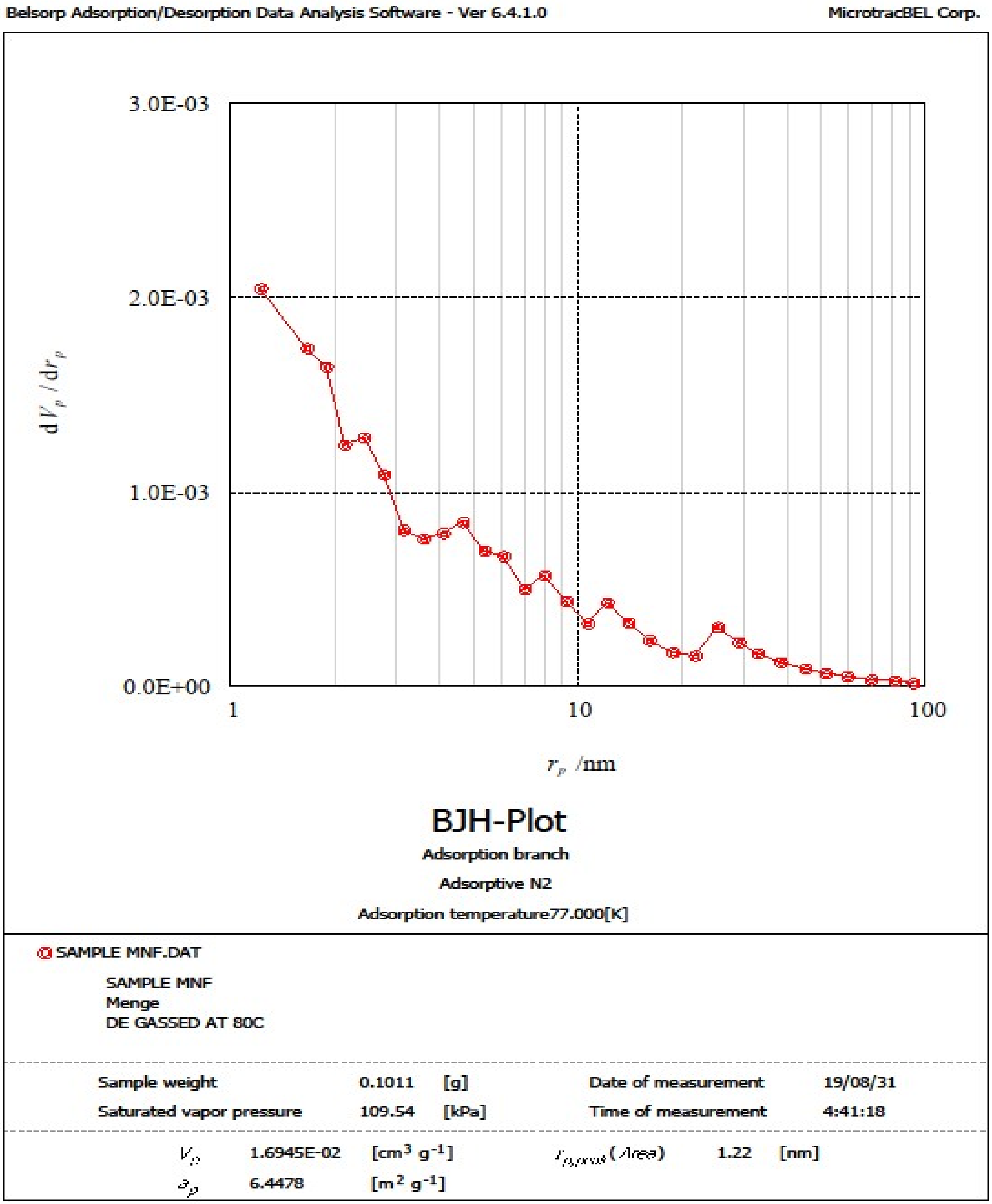

SI. Figure 3 The Barrett-Joyner-Halenda (BHJ) plot of MoS_2 nanosheetnm
